# Isolation, Associated Factors and Antimicrobial Susceptibility Patterns of *Salmonella* spp. in Human Stool, Eggshell Swab and Feces of Egg‐Laying Hens in Mekelle and Southeast Zones of Tigrai, Ethiopia

**DOI:** 10.1002/vms3.71140

**Published:** 2026-08-09

**Authors:** Atsebaha Gebrekidan Kahsay, Tsehaye Asmelash, Enquebaher Kassaye

**Affiliations:** ^1^ Department of Medical Microbiology and Immunology, Faculty of Medical Laboratory Sciences, College of Health Sciences Mekelle University Mekelle Ethiopia; ^2^ Department of Veterinary Public Health and Food Safety, Faculty of Veterinary Sciences, College of Veterinary and Animal Sciences Mekelle University Mekelle Ethiopia

**Keywords:** antimicrobial susceptibility, eggshells, Ethiopia, faeces, *Salmonella* spp, stool, Tigrai

## Abstract

**Background:**

Salmonellosis is an important foodborne and zoonotic disease commonly related to the ingestion of poultry products contaminated with *Salmonella* spp. Although a wide range of poultry farming practices are widely practised in the region, public health concerns regarding the distribution of foodborne zoonotic pathogens at the human–poultry interface remain poorly understood. Therefore, this study aimed to determine the prevalence, associated factors and antimicrobial susceptibility patterns of *Salmonella* spp. at the human‐egg layer farm interface in the Mekelle and southeast zones, Tigrai, Ethiopia.

**Methods:**

A cross‐sectional study was conducted from 1 January to 30 June 2025. Data were collected from poultry farm personnel, and 44 stool samples from farm workers, 44 pooled eggshell swab samples and 44 fresh fecal samples from egg‐laying hens were collected. Standard microbiological procedures, including pre‐enrichment, selective enrichment, selective isolation and biochemical identification, were used to detect suspected *Salmonella* spp. isolates. Antimicrobial susceptibility testing was performed using the Kirby–Bauer disk diffusion method. Data were entered, cleaned and analysed using the Statistical Package for Social Sciences (SPSS) version 23.

**Results:**

Three *Salmonella* spp. were identified from the collected specimens. Of these, 2 (4.55%) were isolated from fecal samples of egg‐laying hens and 1 (2.27%) from pooled eggshell swab samples. However, *Salmonella* spp. were not detected among stool samples collected from farm workers. More than 80% of farm workers reported not practicing hand washing after handling eggs, and 68% lacked knowledge about disease‐causing pathogens that may be transmitted through eggshell contact. No statistically significant association was observed between *Salmonella* spp. occurrence and the assessed risk factors. Antimicrobial susceptibility testing revealed that isolates were resistant to β‐lactams, tetracyclines and aminoglycosides, and two of the three isolates were multidrug resistant.

**Conclusion:**

The detection of *Salmonella* spp., including multidrug‐resistant strains, in egg‐layer farm environments indicates a potential public health concern. Strengthening biosecurity measures, improving hygiene practices, promoting responsible antimicrobial use and implementing continuous One Health surveillance are essential to minimise the risk of pathogen transmission.

## Graphical abstract

Illustration of Possible *Salmonella* spp. Contamination Pathways among the Animal‐Food‐Human‐Environment Interface at the Poultry Farm Settings. Source: Author's illustration.

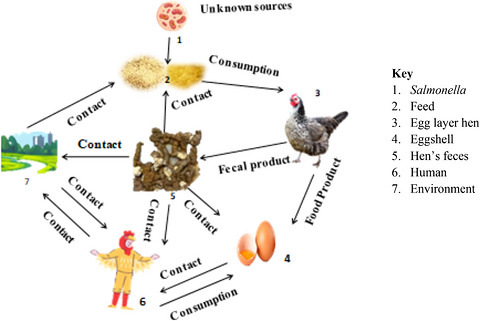

## 1 Introduction

Global egg production and consumption have increased substantially, rising from 14 to 1633 million tons between 1961 and 2021, with China, India, Indonesia and the United States being the leading producers (Solis et al. 2023). Although eggs are an important source of nutrients, food‐producing animals may carry foodborne pathogens even when apparently healthy, creating public health risks if production and handling practices are inadequate (European Food Safety Authority 2025). Eggs may become contaminated through faecal shedding, contaminated feed, insects, handling, storage or trans‐ovarian transmission from infected hens (Singh et al. 2010). Poor hygiene practices during egg handling have also been associated with an increased risk of *Salmonella* spp. transmission (Whiley et al. 2017; Adane et al. 2025). Unsafe food causes substantial health and economic burdens, particularly in low‐ and middle‐income countries (WHO 2022).

Salmonellosis is among the most important foodborne zoonotic diseases worldwide and is commonly associated with poultry, eggs and egg products (ECDC 2024). Contact with poultry farms and eggs has been reported as a risk factor for *Salmonella*‐associated infections (Borchers and Pieler 2010). The disease disproportionately affects vulnerable groups, including children, older adults, pregnant women and immunocompromised individuals and non‐typhoidal *Salmonella* spp. can cause invasive infections such as bacteremia (Plumb et al. 2023).

The occurrence of *Salmonella* spp. in poultry production systems has been documented globally. Studies have reported *Salmonella* spp. contamination in poultry farms, eggshells, poultry carcasses and farm environments in different countries, including China, Brazil, Burkina Faso, Nigeria and Cameroon (Yang et al. 2011; Li et al. 2020; Agbaje et al. 2021; Djuikoue et al. 2023; Brasileiro et al. 2025). In Ethiopia, *Salmonella* spp. prevalence in poultry‐related samples has been reported to be between 2.65% and 20.1%, while detection among poultry‐exposed humans and egg‐laying hen faeces has also been documented (Abdi et al. 2017; Taddese et al. 2019; Tadesse and Ali 2024; Adane et al. 2025).

The emergence of antimicrobial‐resistant *Salmonella* spp. further threatens food safety and public health. High resistance levels to commonly used antimicrobials, including ampicillin, tetracycline, chloramphenicol, streptomycin and sulphamethoxazole‐trimethoprim, have been reported in different countries (Miko et al. 2005; Sodagari et al. 2015; Amajoud et al. 2017). In Ethiopia, resistance exceeding 50% and even 90% has been reported among *Salmonella* spp. isolates, highlighting the need for continuous surveillance and appropriate antimicrobial use (Abdi et al. 2017; Woyessa et al. 2021).

Tigrai has one of the highest levels of poultry ownership in Ethiopia, with more than 7 million poultry, including over 2 million laying hens and annual egg production exceeding 14 million (Federal Democratic Republic of Ethiopia Central Statistical Agency 2021). Despite extensive poultry production and close human–poultry interactions, the burden of foodborne zoonotic pathogens, particularly *Salmonella* spp., at the human‐egg layer farm interface remains poorly characterised in the region. Available studies have largely investigated human or animal sources separately, with limited evidence from integrated assessments involving farm workers, eggs and poultry. Moreover, data on antimicrobial susceptibility patterns of *Salmonella* spp. circulating within egg‐laying farms are scarce. Therefore, this study aimed to determine the prevalence, associated risk factors and antimicrobial susceptibility patterns of *Salmonella* spp. at the human‐egg layer farm interface in the Mekelle and southeast zones, Tigrai, Ethiopia.

## 2 Materials and Methods

### 2.1 Study Area, Design and Period

A farm‐based cross‐sectional study was conducted from January to June 2025 among egg‐layer farms in Mekelle City and the southeast zone of Tigrai Regional State, Northern Ethiopia. Mekelle is the capital city of Tigrai Regional State and is located approximately 784 km north of Addis Ababa, the capital city of Ethiopia. The study area lies between 12°14′50.50″ N to 14°53′48.03″ N latitude and 36°26′48.74″ E to 39°59′00.09″ E longitude. Tigrai has a substantial poultry production system, with more than 7 million poultry, including over 2 million laying hens and annual egg production exceeding 14 million eggs (Federal Democratic Republic of Ethiopia Central Statistical Agency 2021). The study sites were selected in three districts of Mekelle City (Ayder, Hawelti and Semen) and two town administrations of the southeast zone of Tigrai (Adigudem and Hagereselam) based on feasibility considerations, including available resources, budget constraints, accessibility and logistical capacity for sample collection and laboratory processing. These areas also had accessible egg‐layer farms and regular human–poultry interactions, allowing assessment of *Salmonella* spp. occurrence at the human‐egg layer farm interface.

### 2.2 Sample Size Determination

The sample size was calculated using the single population proportion formula based on previously reported *Salmonella* spp. prevalence among eggshell samples (12.2%), egg‐layer fecal samples (14.4%) and stool samples from poultry‐exposed humans (4.5%) in Ethiopia (Tadesse and Ali 2024; Waktole et al. 2024; Akalu et al. 2024). A 95% confidence level and 5% margin of error were considered. The calculated sample sizes were 164 eggshell swab samples, 189 egg‐layer faecal samples and 66 human stool samples.

However, during the study period, the number of eligible egg‐layer farms available in the selected study areas was limited. Therefore, all accessible farms fulfilling the inclusion criteria were included in the study. From each farm, one pooled eggshell swab sample (six eggs per pool), one fresh fecal sample from egg‐laying hens and one stool sample from a farm worker were collected, resulting in a total of 132 samples (44 eggshell pools, 44 hen fecal samples and 44 human stool samples). The final sample size was determined by the availability of eligible farms rather than selective exclusion.

### 2.3 Sampling Method

A list of egg‐layer farms was obtained from the respective agricultural offices. Based on this list, the study farms were distributed among the selected study sites, including Ayder, Hawelti, Semen and Adigudem towns. Due to the limited number of eligible farms, all accessible farms that fulfilled the inclusion criteria were included using a convenience sampling approach.

### 2.4 Data Collection Survey

A structured questionnaire was used to collect information on farm characteristics, poultry management practices, hygiene practices, biosecurity measures, antimicrobial use and potential risk factors associated with *Salmonella* spp. occurrence. The questionnaire was prepared in English, translated into Tigrigna and pretested on eight individuals outside the study area to ensure clarity and consistency. Face‐to‐face interviews were conducted with farm workers using the Open Data kit. Farm hygiene practices were also observed and recorded using a checklist. Sample collection was conducted during the same visit.

### 2.5 Sample Collection, Isolation and Identification of *Salmonella* spp

Eggshell samples were collected using sterile cotton swabs. Six eggs from each farm were swabbed and pooled into a sterile container containing 10 mL of buffered peptone water (BPW; HiMedia, India). Approximately 30 g of fresh fecal droppings from egg‐laying hens were collected aseptically. In addition, 2 g of stool samples were collected from farm workers using sterile containers containing Cary–Blair transport medium. All samples were transported in a cold box at approximately 4°C to the Microbiology Laboratory of the College of Health Sciences, Mekelle University, Tigrai, Ethiopia.

Isolation and identification of *Salmonella* spp. from eggshell and fecal samples were performed according to ISO 6579‐1 (2017). Samples were pre‐enriched in BPW, enriched in Rappaport–Vassiliadis and Selenite Cysteine broths and cultured on Xylose Lysine Deoxycholate (XLD) agar and Brilliant Green Agar (BGA). Human stool samples were enriched using selenite cysteine broth and cultured on XLD and MacConkey agar.

Suspected *Salmonella* spp. colonies were purified on nutrient agar and identified using biochemical tests, including Triple Sugar Iron agar (TSI), Lysine Iron Agar (LIA), Simmons citrate, urease and Sulphide Indole Motility (SIM) tests. *Salmonella* spp. identification was based on characteristic biochemical reactions according to standard procedures.

### 2.6 Antimicrobial Susceptibility Testing

Antimicrobial susceptibility testing was performed using the Kirby–Bauer disk diffusion method according to Clinical and Laboratory Standards Institute (CLSI) guidelines (CLSI 2020). A bacterial suspension equivalent to 0.5 McFarland turbidity standard was prepared and inoculated onto Mueller–Hinton agar. Antibiotic discs were applied, and plates were incubated at 35°C–37°C for 16–18 h.

The tested antimicrobials included ampicillin, ceftriaxone, cefotaxime, ceftazidime, meropenem, streptomycin, gentamicin, ciprofloxacin, cotrimoxazole, tetracycline, chloramphenicol and amoxicillin‐clavulanic acid. The inhibition zones were measured and interpreted as susceptible, intermediate or resistant according to CLSI breakpoints.

### 2.7 Quality Management and Data Analysis

All laboratory procedures were performed following standard protocols. Culture media were checked for sterility and performance before use. *Escherichia coli* ATCC 25922 and *Salmonella Typhimurium* (ATCC 14028) were used as negative and positive quality control strains, respectively. Data were coded, cleaned, entered into Microsoft Excel and analysed using SPSS version 23 and Stata V 16. Descriptive statistics were used to calculate the prevalence of *Salmonella* spp. in relation to the associated factors.

## 3 Results

### 3.1 Background Characteristics of Egg Layer Farm Workers

A total of 44 egg‐layer farms were included in the study from Mekelle City and the southeast zone of Tigrai Regional State, Ethiopia. The participating farms were located in Ayder (*n* = 16), Hawelti (*n* = 14), Semen (*n* = 9) and Adigudem town (*n* = 5). One farm worker who had direct contact with egg‐layer farm activities was included from each farm for questionnaire administration and stool sample collection. Nearly half of the farm workers were aged between 26 and 35 years. Females accounted for 54.5% of the participants. Approximately three‐fourths of the participants had completed primary or secondary education, and more than 65% were married. Six participants reported experiencing abdominal cramps within the two weeks prior to the study period. Regarding stool characteristics, 61% of the collected stool samples were loose in consistency (Table 1).

**TABLE 1:** Background characteristics of egg laying farm workers in the Mekelle and southeast zones of Tigrai, Ethiopia.

Variables	Frequency	Percent
Age in year		
18–25	5	11.4
26–35	21	47.7
36–45	13	29.5
46–55	3	6.8
56–65	2	4.6
> 65	0	0
Gender		
Male	20	45.5
Female	24	54.5
Educational status		
Unable to read and write	7	15.9
Elementary	15	34.1
Secondary	14	31.8
Diploma	3	6.8
Degree	5	11.4
Marital status		
Single	12	27.3
Married	29	65.9
Divorced	3	6.8
Widowed	0	0
Service year of egg layers farm workers		
Up to 10	42	95.5
11‐20	2	4.5
Do you have abdominal cramps in the last 2 weeks		
Yes	6	13.6
No	38	86.4
A type of stool collected from a contact human		
Formed	16	36.3
Loose	27	61.4
Watery	1	2.3
Availability of latrine in the egg layer farms		
Yes	22	50
No	22	50
Do you wash your hands after visiting latrine?		
Yes	32	72.7
No	12	27.3
Sub‐cities/towns of the study area		
Ayder	16	36.4
Semen	14	31.8
Hawelti	9	20.5
Adigudem	5	11.3

### 3.2 Prevalence of *Salmonella* spp. Among Human, Egg‐Laying Hen Faeces and Eggshell

A total of 132 samples, 44 human stool, 44 feces of egg‐laying hens and 44 eggshell swabs of six pooled eggs, were collected. Three *Salmonella* spp. isolates were recovered from the aforementioned samples, including one from eggshells and two from the faeces of egg‐laying hens. *Salmonella* spp. was not recovered from the stool of egg‐layer farm workers (Table 2).

**TABLE 2:** Prevalence of *Salmonella* in human stool, feces of egg‐laying hens and eggshell swabs at farm level in Mekelle and southeast zones of Tigrai, Ethiopia.

Sample type	Hawelti		Ayder		Semen		Adigudem		Total	
	Pos	Neg	Pos	Neg	Pos	Neg	Pos	Neg	Pos	Neg
Stool	0	44	0	44	0	44	0	44	0	44
Feces	1	44	1	43	0	43	0	44	2	42
Eggshells	1	43	0	44	0	44	0	44	1	43
Total	2	130	1	131	0	132	0	132	3	129

### 3.3 Factors Associated With *Salmonella* spp

The association between *Salmonella* spp. detection from eggshell swab samples and potential farm‐level risk factors was assessed using Fisher's exact test due to the low number of positive samples. More than 80% of the farm workers reported that they did not routinely wash their hands after handling eggs. In addition, 68% of participants were not aware that eggshells could harbour disease‐causing microorganisms. Consumption of raw or undercooked egg‐containing foods was reported by less than 7% of participants. Approximately 90% of farms reported that farm owners were responsible for overseeing hygiene practices. However, more than 80% of farms reported that they did not have established hygiene protocols or written procedures to prevent bacterial contamination. No statistically significant association was observed between *Salmonella* spp. detection in eggshell swabs and the assessed farm‐level risk factors (Table 3).

**TABLE 3:** Cross‐tabulation of *Salmonella* in eggshell swabs with farm‐level risk factors in the Mekelle and southeast zones of Tigrai, Ethiopia.

Variables	*Salmonella* isolates			*p*
	Pos, *N* (%)	Neg, *N* (%)	Total, *N* (%)	
Do you wash your hands after handling eggs?				0.455
Yes	0 (0)	8 (100)	8 (18.2)	
No	1 (2.78)	35 (97.22)	36 (81.8)	
How do you wash your hands after handling eggs?				0.705
Soap and cold water	0 (0)	13 (100)	13 (29.5)	
Cold water only	1 (3.2)	30 (96.8)	31 (70.5)	
Do you take food after handling eggs and before washing your hands?				0.523
Yes	0 (0)	22 (100)	22 (50)	
No	1 (4.5)	21 (95.5)	22 (50)	
How do you handle eggs in the farm?				0.889
Bare hands	1 (2.6)	38 (97.49)	39 (88.6.)	
Using glove	0(00)	5 (92.9)	5 (11.4)	
Do you clean eggs before selling or storage?				0.591
Yes	0 (0)	18 (100)	18 (40.9)	
No	1 (3.8)	25 (96.2)	26 (59.1)	
Did you take training about food safety?				0.636
Yes	0 (0)	16 (100)	16 (36.4)	
No	1 (3.7)	27 (97.6)	28 (63.6)	
Do you think eggshells contain disease causing bacteria				0.682
Yes	0 (0)	14 (100)	14 (31.8)	
No	1 (2.4)	29 (97.6)	30 (68.2)	
Do you consume foods containing raw or undercooked				0.932
Yes	0 (0)	3 (100)	3 (6.8)	
No	1 (2.4)	40 (97.5)	41 (93.2)	
Do you have a specific hygiene protocol in place to prevent bacterial contamination?				0.841
Yes	0 (0)	7 (100)	7 (15.9)	
No	1 (2.8)	36 (97.2)	37 (84.1)	
Who is responsible for overseeing hygiene practices on the farm				0.909
Farm owner	1 (2.5)	39 (7.5)	40 (90.9)	
Farm manager	0 (0)	4 (100)	4 (9.1)	

### 3.4 Factors Associated With *Salmonella* spp

Egg‐layer farms established between 2001 and 6 months before the study period were included. Nearly 80% of the participating farms were established between 2011 and 2020, and both *Salmonella* spp. isolates were recovered from farms within this establishment period. More than three‐fourths of the farms were categorised as closed‐type farms, and both detected *Salmonella* spp. isolates were obtained from this category. Only approximately 11% of the farms had more than 500 egg‐laying hens, and no *Salmonella* spp. isolate was detected from these farms. Regarding the knowledge of farm workers, approximately 84% of respondents reported that contact with egg‐layer faeces does not cause infection, and both *Salmonella* spp. isolates were detected among farms in this group. Half of the respondents reported routine antibiotic use for the prevention of infection in egg‐laying hens, with one *Salmonella* spp. isolate detected among these farms. Less than 10% of farms reported a previous occurrence of disease outbreaks during the farm's history (Table 4).

**TABLE 4:** Cross‐tabulation of *Salmonella* in faeces of egg layer hens with farm‐level risk factors in Mekelle and southeast zones of Tigrai, North Ethiopia.

Variables	*Salmonella* isolates			*p*
	Pos, *N* (%)	Neg, *N* (%)	Total, *N* (%)	
Establishment year of egg layer farms				0.827
2001–2010	0 (0)	6 (100)	6 (13.6)	
2011–2020	2 (5.7)	33 (94.3)	35 (79.5)	
> 2020	0 (0)	3 (100)	3 (6.9)	
Type of egg layer farms				0.460
Open	0 (0)	14 (100)	14 (31.8)	
Closed	2 (6.7)	28 (93.3)	30 (68.2)	
Egg layer farm size				0.451
Up to 50	0 (0)	14 (100)	14 (31.8)	
51–500	2 (8)	23 (92)	25 (56.8)	
> 500	0 (0)	5 (100)	5 (11.4)	
Type of faeces collected from the egg layer farms				0.492
Formed	0 (0)	13 (100)	13 (29.4)	
Loose	2 (6.5)	29 (93.5)	31 (70.6)	
Watery	0 (0)	0 (0)	0 (0)	
Do you think contact with faeces of egg layers acquire infection?				0.704
Yes	0 (0)	20 (100)	20 (45.5)	
No	2 (5.4)	22 (94.5)	24 (54.5)	
How often egg layer hens are screened for infection				0.292
Only when symptoms appear	0 (0)	17 (100)	17 (38.6)	
Never	1 (6.7)	14 (93.3)	15 (34.1)	
Regularly	1 (9)	10 (91)	11 (25)	
Annually	0 (0)	1 (100)	1 (2.3)	
Do you isolate sick or potentially infected hens from the rest of the herd/flock?				0.708
Yes	1 (4.2)	23 (95.8)	24 (54.5)	
No	1 (5)	19 (5)	20 (45.5)	
Are antibiotics used routinely to prevent infection				0.756
Yes	1 (4.5)	21 (95.5)	22 (50)	
No	1 (4.5)	21 (95.5)	22 (50)	
Have you experienced any infection outbreaks on your farm in the past 12 months?				0.825
Yes	0 (0)	4 (100)	4 (9.1)	
No	2 (5)	38 (95)	40 (90.9)	

### 3.5 Antimicrobial Susceptibility Patterns of *Salmonella* spp

Of the twelve antimicrobial discs representing nine antimicrobial classes tested, the two *Salmonella* spp. isolates recovered from egg‐laying hen feces and the single isolate from eggshell swabs showed susceptibility to carbapenems, cephalosporins, sulphonamides and fluoroquinolones. In contrast, all isolates demonstrated resistance to β‐lactams, tetracyclines and aminoglycosides (Table 5).

**TABLE 5:** Antimicrobial susceptibility patterns of *Salmonella* from egg shell swabs and feces of egg laying hens at the farm level in the Mekelle and southeast zones of Tigrai, Ethiopia.

Antimicrobial class categories	Antimicrobial susceptibility patterns		*Salmonella* isolates	
			Egg layer faeces, *N* (%)	Eggshell swabs, *N* (%)
Carbapenems	Meropenem	Resistance	0 (0)	0 (0)
		Sensitive	2 (100)	1 (100)
Cephalosporins	Ceftriaxone	Resistance	0 (0)	0 (0)
		Sensitive	2 (100)	1 (100)
	Cefotaxime	Resistance	0 (0)	0 (0)
		Sensitive	2 (100)	1 (100)
	Ceftazidime	Resistance	0 (0)	0 (0)
		Sensitive	2 (100)	1 (100)
B‐lactams	Ampicillin	Resistance	1 (50)	1 (100)
		Sensitive	1 (50)	0 (0)
	Amoxicillin‐clavulanic acid	Resistance	1 (50)	1 (100)
		Sensitive	1 (50)	0 (0)
Sulphonamides	Cotrimoxazole	Resistance	0 (0)	0 (0)
		Sensitive	2 (100)	1 (100)
Fluoroquinolones	Ciprofloxacin	Resistance	0 (0)	0 (0)
		Sensitive	2 (100)	1 (100)
Amphenicoles	Chloramphenicol	Resistance	1 (50)	0 (0)
		Sensitive	1 (50)	1 (100)
Tetracycline	Tetracycline	Resistance	1 (50)	1 (100)
		Sensitive	1 (50)	0 (0)
Aminoglycosides	Streptomycin	Resistance	1 (50)	1 (100)
		Sensitive	1 (50)	0 (0)
	Gentamicin	Resistance	1 (50)	0 (0)
		Sensitive	1 (50)	1 (100)

### 3.6 Multidrug‐Resistant Isolates of *Salmonella* spp

One of the two *Salmonella* spp. isolates recovered from egg‐laying hen fecal samples and the single isolate from eggshell swabs exhibited multidrug resistance. Each multidrug‐resistant isolate showed resistance to at least one antimicrobial agents in three classes of antimicrobials (, as presented in Table 6.

**TABLE 6:** Multidrug resistance patterns of *Salmonella* isolated from feces and eggshell swabs from egg layers in Mekelle and southeast zones of Tigrai, Ethiopia.

Antimicrobials	Isolates	Antimicrobial classes	MDR pattern, *N* (%)
Egg layer feces			
C, TET, ST	1	3	1 (50)
AMP/AMC, GEN	1	2	
Eggshell swabs			
AMP/AMC, TET, ST	1	3	1 (100)

Abbreviations: AMC, amoxicillin‐clavulanic acid; AMP, ampicillin; C, chloramphenicol; G, gentamicin; ST, streptomycin; TET, tetracycline.

## 4 Discussion

Salmonellosis is an important foodborne and zoonotic disease that is commonly associated with the consumption of poultry products contaminated with *Salmonella* (Ehuwa et al. 2021). According to reports, the Tigrai region has the largest poultry population in Ethiopia (Hirvonen et al. 2020). Such a high poultry density may pose a significant public health risk if poultry–human interactions are not properly managed. In the present study, *Salmonella* was detected in 2.27% of eggshell samples collected from egg‐laying farms. This prevalence is lower than reports from other parts of Ethiopia (4.8%) (Taddese et al. 2019), (4.5%) (Woyessa et al. 2021), (12.2%) (Tadesse and Ali 2024), (21.4%) (Adane et al. 2025), 15.38% (Nguse et al. 2015), Nigeria (13%) (Agbaje et al. 2021), Cameroon (23%) (Djuikoue et al. 2023) and India (3.84%) (Singh et al. 2010). Nevertheless, our finding was higher than findings from eggshell swab samples in Ethiopia (0%) (Taddese et al. 2019) and France (1.05%) (Chemaly et al. 2009). The observed difference may be due to variations in poultry management systems, biosecurity practices, hygiene standards, sampling strategies, laboratory techniques and geographic or environmental factors. Although variations in *Salmonella* prevalence have been observed across studies conducted both in Ethiopia and other countries, the consumption of egg‐based food products contaminated with *Salmonella* remains a significant cause of foodborne outbreaks, as documented in Slovakia, Spain and Poland (Ehuwa et al. 2021). The detection of *Salmonella* on eggshells indicates contamination at the farm or environmental level and represents a potential risk to farm workers, consumers and the poultry industry as a whole.

On the other hand, *Salmonella* was isolated from the fresh faeces of 4.54% of egg‐laying farms. This was similar to a study in Ethiopia (4.7%) (Eguale 2018) but slightly higher than another study in Ethiopia (2.4%) (Taddese et al. 2019). However, our result was lower than studies in Ethiopia (18.4%) (Woyessa et al. 2021), (7.14%) (Akalu et al. 2024), (20.2%) (Waktole et al. 2024), Western Australia (54.5%) (Sodagari et al. 2020) and Uganda (20.7%) (Odoch et al. 2017). The presence of *Salmonella* in the faeces of egg‐laying suggests a potential direct impact on farm workers, especially under conditions of inadequate hygiene (Delahoy et al. 2018). In this study, *Salmonella* was not identified in the stool of egg‐laying farm workers. This was in line with a study reported from Ethiopia (0%) (Adane et al. 2025), although *Salmonella* was detected from the stool (4.5%) (Akalu et al. 2024) and hand swabs of egg‐laying (4.5%) (Woyessa et al. 2021) farm workers in Ethiopia. The variation may be different in sample size, farm management, method and seasonal variation (Pacholewicz et al. 2023).

In the present study, none of the assessed eggshell‐related risk factors showed a statistically significant association with *Salmonella* isolation. However, questionnaire‐based data revealed important gaps in food safety and hygiene practices among farm workers. Some workers reported inadequate hand washing after handling eggs, limited awareness that eggshells may harbour pathogenic microorganisms and poor knowledge regarding the risks associated with the consumption of raw or undercooked foods. Approximately two‐thirds of the farm workers had not received food safety training. In addition, no statistically significant association was observed between *Salmonella* positivity and farm size, year of establishment, farm type or contact with hen faeces. Although significant associations were not identified in this study, poor hygiene practices and weak biosecurity measures are known to increase the risk of *Salmonella* contamination in the farm environment and egg‐laying production systems, as reported by previous studies elsewhere (Kabeta et al. 2024). Moreover, a lack of effective cleaning and disinfection, as well as a lack of training, is linked to *Salmonella* infection (Mdemu et al. 2025).

All of the *Salmonella* isolates from the faeces and eggshell swabs were susceptible to carbapenem, ceftazidime, cefotaxime, ceftriaxone, cotrimoxazole and ciprofloxacin. Resistance to amoxicillin/clavulanic acid, ampicillin, streptomycin and tetracycline was reported. This result is supported by previous studies from Ethiopia (Abdi et al. 2017; Akalu et al. 2024; Tadesse and Ali 2024) and China (Li et al. 2020). The only isolate recovered from the eggshells showed MDR to ampicillin, amoxicillin/clavulanic acid, tetracycline and streptomycin, which is supported by studies from Ethiopia (Abdi et al. 2017), Uganda (Odoch et al. 2017) and China (Li et al. 2020).

The microbiological analysis of eggshell swabs, fresh faecal droppings and stool samples in egg‐laying farms, together with the assessment of risk factors and antimicrobial susceptibility testing, provides valuable insights for food safety and the One Health approach. This was considered the strength of this study. However, this study has some limitations, including a relatively small sample size and low isolation rates of *Salmonella*. In addition, the study did not include serotyping or molecular characterisation of the isolates, which could have provided a more detailed understanding of strain diversity and transmission dynamics. Moreover, the achieved sample size was smaller than the calculated requirement; therefore, we interpreted the result as preliminary and exploratory, with acknowledgement of the reduced statistical power.

## 5 Conclusion and Recommendation

*Salmonella* was detected at a low prevalence in faecal and eggshell samples from egg‐laying farms, with no detection in farm worker stool. Although the prevalence was low compared with other studies, the presence of *Salmonella*, including multidrug‐resistant strains, is of significant public health concern, as food products should be free of this pathogen. These results underscore the importance of a One Health approach including comprehensive biosecurity measures, responsible antimicrobial use and ongoing surveillance to prevent contamination, reduce the spread of resistant bacteria, and safeguard both animal and human health.

## Author Contributions

**Enquebaher Kassaye**: conceptualisation, investigation, writing – review and editing, validation, methodology, supervision, data curation. **Atsebaha Gebrekidan Kahsay**: investigation, conceptualisation, writing – original draft, methodology, validation, visualisation, writing – review and editing, data curation, project administration, formal analysis. **Tsehaye Asmelash**: conceptualisation, investigation, writing – review and editing, methodology, validation, supervision, formal analysis.

## Funding

We received a Partial Fund from Institutional Collaboration Program between Hawassa and Mekelle Universities of Ethiopia and the Norwegian University of Life Sciences, ICP V NORAD Project with a fund number of SS0037/24.

## Ethics Statement

This study received ethical clearance from the Institutional Review Board (IRB) (MU‐IRB 2439/2024) of Mekelle University, College of Health Sciences. The study was carried out in accordance with relevant national, international and scientific guidelines, in accordance with the Declaration of Helsinki.

## Consent

Informed consent was collected from participants and guardians (unable to read and write) of the egg layer farm workers during the study period.

## Conflicts of Interest

The authors declare no conflicts of interest.

## Data Availability

The data that support the findings of this study are available from the corresponding author upon reasonable request.
